# Генетические аспекты устойчивости картофеля к фитофторозу

**DOI:** 10.18699/VJ21.020

**Published:** 2021-03

**Authors:** T.S. Frolova, V.A. Cherenko, O.I. Sinitsyna, A.V. Kochetov

**Affiliations:** Institute of Cytology and Genetics of the Siberian Branch of the Russian Academy of Sciences, Novosibirsk, Russia Novosibirsk State University, Novosibirsk, Russia; Institute of Cytology and Genetics of the Siberian Branch of the Russian Academy of Sciences, Novosibirsk, Russia Novosibirsk State University, Novosibirsk, Russia; Institute of Cytology and Genetics of the Siberian Branch of the Russian Academy of Sciences, Novosibirsk, Russia Novosibirsk State University, Novosibirsk, Russia; Institute of Cytology and Genetics of the Siberian Branch of the Russian Academy of Sciences, Novosibirsk, Russia Novosibirsk State University, Novosibirsk, Russia

**Keywords:** Phytophthora infestans, Solanum tuberosum, late blight, resistance, R-genes, quantitative trait loci, secondary metabolites, Phytophthora infestans, Solanum tuberosum, фитофтороз, резистентность, R-гены, локусы количественных признаков, вторичные метаболиты

## Abstract

Оомицет Рhytophthora infestans Mont. de Bary – основной патоген сельскохозяйственных культур семейства Пасленовые, особенно картофеля (Solanum tuberosum). С учетом того, что картофель – четвертая культура в мире по масштабам выращивания, ежегодные потери от фитофтороза огромны. Исследования базовых механизмов взаимодействия между картофелем и возбудителем фитофтороза не только расширяют фундаментальные знания в этой области, но и открывают новые возможности для влияния на эти взаимодействия с целью повышения резистентности к патогену. Взаимодействие картофеля и возбудителя фитофтороза можно рассматривать с генетической точки зрения, причем интересны как ответ картофеля на процесс колонизации со стороны P. infestans, так и изменение активности генов у фитофторы при заражении растения. Можно исследовать этот процесс через изменение профиля вторичных метаболитов хозяина и патогена. Помимо фундаментальных исследований в этой области, не меньшее значение имеют и прикладные работы в виде создания новых препаратов для защиты картофеля. Представленный обзор кратко описывает основные этапы исследований устойчивости картофеля к фитофторозу, начиная с самых первых работ. Большое внимание уделяется ключевым моментам по изменению профиля вторичных метаболитов (фитоалексинов). Отдельный раздел посвящен описанию как качественных, так количественных признаков устойчивости картофеля к возбудителю фитофтороза: их вкладу в общую резистентность, картированию и возможности регуляции. Оба вида признаков важны для селекции картофеля: качественная устойчивость за счет R-генов быстро преодолевается патогеном, в то время как пирамидирование локусов количественных признаков способствует созданию высокоустойчивых сортов. Новейшие подходы молекулярной биологии дают возможность изучать и транслятомные профили, что позволяет посмотреть на взаимодействие картофеля и возбудителя фитофтороза. Показано, что процесс колонизации картофеля отражается не только на активности различных генов и профиле вторичных метаболитов, выявлены также белки-маркеры ответа на заражение со стороны картофеля – это патоген-зависимые белки и пластидная углекислая ангидраза. Маркерами заражения от P. infestans были белки грибной целлюлозо-синтазы и гаусторий-специфический мембранный белок. В данном обзоре приведена информация по наиболее актуальным комплексным исследованиям генетических механизмов устойчивости картофеля к фитофторозу.

## Abstract (en)

Phytophthora infestans Mont. de Bary is the main oomycete pathogen of cultivated crops in the family Solanaceae, especially potato (Solanum tuberosum). Because potato is the fourth most cultivated crop worldwide, its annual losses from late blight are tremendous. Studies of the basic mechanisms of interaction between potato and the late blight pathogen not only expand the fundamental knowledge in this area, but also open up new possibilities for regulating these interactions in order to increase resistance to the pathogen. The interaction of potato and the late blight pathogen can be considered from a genetic point of view, and it is interesting to consider both the response of the potato to the colonization process by P. infestans and the change in gene activity in late blight during plant infection. We can also investigate this process by changing the profile of secondary metabolites of the host and the pathogen. In addition to fundamental work in this area, applied work in the form of the development of new preparations for protecting potatoes is of no less importance. This review briefly describes the main stages of studies of potato resistance to late blight, starting almost from the first works. Much attention is paid to key works on changing the profile of secondary metabolites phytoalexins. A separate section is devoted to the description of both qualitative and quantitative characteristics of potato resistance to the late blight pathogen: their contribution to overall resistance, gene mapping, and regulation capabilities. Both types of traits are important for potato breeding: quantitative resistance due to R-genes is quickly overcome by the pathogen, while quantitative trait loci make it possible to create varieties with almost absolute resistance due to the pyramid of effective genes. The latest approaches in molecular biology make it possible to study translatomic profiles, which makes it possible to look at the interaction of potatoes and the late blight pathogen at a different angle. It has been shown that the process of potato colonization affects not only the activity of various genes and the profile of secondary metabolites: proteins-markers of the response to infection from potatoes have also been identified: they are pathogen-bound proteins and plastid carbonic anhydrase. On the part of P. infestans, fungal cellulose synthase proteins and haustorium-specific membrane protein were markers of infection. Thus, the review contains information on the most relevant complex studies of the genetic mechanisms of potato resistance to late blight.

## Введение

Картофель, или паслен клубненосный (Solanum tuberosum L.), был одомашнен около 7000–10 000 лет назад на территории современного Южного Перу. В Европу картофель был завезен испанцами относительно недавно, во второй половине XVI в. С тех пор он стал одной из основных сельскохозяйственных культур, занимая по масштабам выращивания четвертое место после кукурузы, пшеницы и ржи. В связи с большими объемами возделывания весьма существенными являются потери урожая из-за различных патогенов, основной из которых – возбудитель фитофтороза (Phytophthora infestans), способного полностью уничтожить растения спустя несколько дней после появления первых симптомов заражения. Поэтому изучение механизмов взаимодействия картофеля и возбудителя фитофтороза на молекулярном и генетическом уровнях очень важны для создания новых подходов для повышения резистентности картофеля.

Первые труды, посвященные взаимодействию S. tuberosum и P. infestans, относятся к концу 1960-х гг. (Ingram, Robertson, 1965; Ingram, 1967; Robertson et al., 1968). В работах проводили сравнение восприимчивого (Majestic) и устойчивого (Orion) к возбудителю фитофтороза сортов картофеля. Ранее было показано, что гены устойчивости (R-гены) экспрессируются только во фрагментах ткани толщиной более 10 клеток (Tomiyama et al., 1958), поэтому исследование осуществляли на клеточных культурах и тканевых агрегатах. Было сделано два ключевых вывода: 1) в тканях обоих сортов содержатся вещества, стимулирующие рост возбудителя фитофтороза, т. е. они являются нормальными метаболитами живых тканей, а не образуются в ответ на заражение; 2) тканевые агрегаты сорта Orion тормозили развитие возбудителя фитофтороза, но после заморозки и, как следствие, разрушения тканей это свойство пропадало. Таким образом, был сделан вывод, что резистентность – это свойство живых тканей, способных реагировать на патоген (Ingram, Robertson, 1965). В дальнейшем было установлено, что картофель сорта Orion может быстро развивать постинфекционную токсичность, тормозящую рост зародышевых трубок P. infestans, тем самым предотвращая инфицирование. Ключевая роль в развитии этого ответа отводится R-гену (Ingram, 1967).

Соединения, обуславливающие устойчивость растений к патогенам, ранее были названы фитоалексинами (Müller, Behr, 1949). В предыдущих исследованиях высказывалось предположение, что именно они нарабатываются в тканях в ответ на инфицирование, что и приводит к ингибированию патогенов. Позднее была опубликована статья, свидетельствующая о накоплении фенольных соединений в токсичных для фитофторы фракциях (Robertson et al., 1968): салициловой, п-гидроксибензойной и ванилиновой кислот. Авторы высказывают предположение, что система устойчивости, основанная на R-гене, может быть связана с постинфекционной индукцией синтеза фитоалексинов. Примерно в это же время были описаны фитоалексины сесквитерпеноидной структуры: ришитин, любимин, фитуберин и солаветивон, выделенные из зараженных клубней картофеля (Katsui et al., 1968; Kuć, 1982; Kuć, Rush, 1985).

Спустя почти 20 лет было издано подробное исследование временного накопления ришитина как наиболее показательного маркера иммунного ответа в клубнях резистентных и восприимчивых сортов в ответ на заражение P. infestans (Rohwer et al., 1987). Обнаружено, что ришитин и некоторые его структурно родственные производные (любимин) быстро накапливались в клубнях при несовместимых взаимодействиях картофеля и фитофторы и достаточно медленно – у совместимых, в листьях подобный ответ отсутствовал. Сделан вывод, что сесквитерпены могут быть полезны в развитии иммунного ответа, но не являются обязательными компонентами устойчивости.

Интересная работа была проведена по приданию устойчивости картофеля к P. infestans через создание соматических и половых гибридов культивируемого S. tuberosum с диким подвидом S. circaeifolium Bitter, устойчивость которого к возбудителю фитофтороза – весьма привлекательный признак для включения в генофонд культурного картофеля. Путем слияния клеток были получены тетраплоидные гибридные каллусы (Mattheij et al., 1992), а растения из них обладали полной устойчивостью к P. infestans. Исследователи сравнили содержание гликоалкалоидов у родительских растений и гибридов и обнаружили повышенное содержание томатидин гликозида, позаимствованное гибридом от дикого S. circaeifolium. Кроме того, был обнаружен новый гликозид демиссидин, не найденный ни у одного из родителей. Отмечаются фертильность полученных женских растений и возможность их скрещивания с культивируемым S. tuberosum для закрепления приобретенной устойчивости. Этой же группой исследователей были созданы половые гибриды S. tuberosum и S. circaeifolium (Louwes et al., 1992). Среди гибридов наблюдались преимущественно триплоидные растения (83 %), несущие двойной геном S. circaeifolium, что делает их более устойчивыми к патогену и, как следствие, более перспективными для использования в селекции. Половые гибриды также обладали повышенным содержанием томатидин гликозида и демиссидин гликозидом, не обнаруженным у родительских растений.

## Картирование признаков устойчивости в геноме S. tuberosum

**Качественные признаки, обусловленные R-генами**

В начале XXI в. развитие компьютерных технологий и статистических методов дало возможность проводить более масштабный анализ групп сцепления, выявлять локусы с генами количественных признаков и строить для них карты сцепления. Были созданы карты сцепления для диплоидного картофеля, согласно которым локусы качественной устойчивости к возбудителю фитофтороза располагаются практически в каждой хромосоме, а на хромосомах III, IV, V и VI были сцеплены с поздней спелостью (Gebhardt, Valkonen, 2001; Simko, 2002). Позднее подобные результаты были получены и для тетраплоидного картофеля (Bradshaw et al., 2004) при скрещивании устойчивого (Stirling) и восприимчивого сортов. Среди множества групп сцепления обнаружен перспективный локус в хромосоме IV, который не сцеплен с поздней спелостью клубней. У устойчивого родительского растения R-ген, переданный потомству, был картирован в хромосоме XI.

Несмотря на обилие работ, посвященных картированию R-генов в геноме картофеля (Ballvora et al., 2002; Van Der Vossen et al., 2003; Park et al., 2005; Restrepo et al., 2005; Bradshaw et al., 2006a, b; Solomon-Blackburn et al., 2007; Brugmans et al., 2008; Tan et al., 2008; Rauscher et al., 2010), следует отметить, что устойчивость к возбудителю фитофтороза сортов картофеля на основании R-генов сохраняется на протяжении 5–10 лет, после чего сорт становится восприимчивым к новым расам P. infestans (Stewart et al., 2003). Распознавание патогена R-геном довольно быстро нивелируется мутациями в соответствующем гене авирулентности P. infestans, что позволяет патогену успешно проникать и колонизировать растение-хозяина при совместимом взаимодействии (Poland et al., 2009).

Биоинформатические методы были использованы и для изучения механизмов восприимчивости. Показано, что карбоангидраза – фермент, обратимо конвертирующий диоксид углерода в бикарбонат, может играть большую роль во время несовместимых взаимодействий между патогеном и хозяином (Restrepo et al., 2005). С помощью ДНК-микрочипов было исследовано временное изменение экспрессии R-генов: в первые часы после заражения (6–12 ч) в целом наблюдалась индукция экспрессии, но спустя некоторое время (48–72 ч) большая часть генов подверглась репрессии. Интересным выглядит подавление жасмонатного пути при восприимчивых взаимодействиях. Однако при заражении в основном подавляются гены, связанные с фотосинтезом: так, наиболее выраженное подавление обнаружено для пластидной карбоангидразы, которая обладает антиоксидантной активностью и способностью связывать салициловую кислоту (Slaymaker et al., 2002). В первые 12 ч наблюдалось существенное усиление экспрессии карбоангидразы при несовместимых взаимодействиях, тогда как через 24 или 48 ч ее следы едва обнаруживались, что позволяло однозначно отличить устойчивые формы. Значительная роль салициловой кислоты именно в раннем ответе S. tuberosum на инфицирование была продемонстрирована с помощью трансгенных NahG растений, которые не способны накапливать салициловую кислоту, их восприимчивость к P. infestans была гораздо выше, чем у дикой формы. Однако предварительная обработка растений препаратом салициловой кислоты практически уравнивала вероятность заражения (Halim et al., 2007).

Помимо единичных доминантных R-генов устойчивости, отвечающих за распознавание соответствующего гена авирулентности P. infestans и запускающих защитный ответ, проявляющийся в локальной гибели клеток (реакция сверхчувствительности) и тем самым останавливающий рост патогенных микроорганизмов, у растений существует группа генов с другим механизмом защиты – гены множественной устойчивости. Исследована экспрессия четырех генов-транспортеров у картофеля, транскрипция которых регулировалась различными препаратами (Ruocco et al., 2011). Среди них были выявлены и те, экспрессия которых существенно возрастает при заражении P. infestans, – гены StPDR1 и StPDR2 экспрессировались активнее в 13 и 37 раз соответственно спустя 18 ч после заражения. Авторы полагают, что все исследованные ими гены (StPDR1–4) являются частью более сложного системного ответа растения на биотические и абиотические факторы.

**Локусы количественных признаков у S. tuberosum**

Масштабная попытка картирования локусов количественных признаков у картофеля была предпринята в 2018 г. (Santa et al., 2018). Исследователи снова выбрали в качестве объекта тетраплоидный геном картофеля, отмечая его высокую важность для селекции и при этом существенные затруднения в силу высокой гетерозиготности у автотетраплоидного картофеля. Ученым удалось обнаружить два новых QTL на хромосомах III и VIII. Отмечается, что один из аллелей первого локуса может опосредовать в среднем более высокую степень тяжести заболевания. Этот локус также включает транскрипционный фактор Arf 2, связанный со старением листьев, вызванный окислительным стрессом у Arabidopsis и передачей сигналов гиббереллина и брассиностероидных путей при взаимодействии растения с патогеном (Vert et al., 2008; Lim et al., 2010; Koch et al., 2016). Аллель, определяющий в среднем более низкую степень тяжести заболевания, содержал QTL хромосомы VIII. Этот маркер связан с геном, который кодирует фактор транскрипции спираль-петля-спираль (bHLH) JAF13, участвующий в биосинтезе флавоноидов у Petunia×hybrida (Quattrocchio et al., 2006).

Были получены трансгенные растения со сверхэкспрессией гена редуктазы D-галактуроновой кислоты и повышенным уровнем L-аскорбата (Chung et al., 2019). После заражения размер некротических пятен у трансгенных растений был меньше, чем в контрольной группе, при этом обнаружено увеличение экспрессии генов, участвующих в антиоксидантной активности. В результате выявлено снижение активных форм кислорода (АФК) в клетках, что может объяснять меньший размер некротического пятна за счет уменьшения ответа реакции сверхчувствительности, индуцируемого АФК. В целом отмечалась большая устойчивость трансгенных растений к возбудителю фитофтороза, однако полной устойчивости не наблюдалось. L-аскорбат снизил содержание абсцизовой кислоты и увеличил содержание гибберелиновой кислоты. Но при этом установлена существенная потеря в урожайности картофеля, которая все же превышала урожай от зараженного.

## Транскриптомный анализ для совместимых и несовместимых взаимодействий P. infestans и S. tuberosum

В 2012 г. впервые выполнен транскриптомный анализ для совместимых и несовместимых взаимодействий P. infestans и S. tuberosum (Gyetvai et al., 2012). Исследовано пять изогенных линий, которые различались только наличием и отсутствием R-гена (R1), с целью исключить влияние генетического фона на транскриптом. Однако оказалось, что изменения транскриптома между растениями одной линии отличаются больше, чем средние транскриптомы линий между собой, что указывает на значительное влияние как индивидуальных физиологических параметров, так и факторов окружающей среды в процессе защитной реакции. Одним из них может быть движение во времени и пространстве защитных сигналов от места первоначального физического контакта между зооспорами P. infestans и клетками-хозяевами и соседними клетками. Анализ DeepSAGE позволил выявить некоторые интересные аспекты общей структуры транскриптома. Две трети (68 %) унитагов были экспрессированы на низких уровнях (<10 CPM) в ткани листьев, из которых одна треть (36 %) не соответствовала ни одному из известных транскриптов картофеля, что может быть объяснено немодельным объектом и немногочисленными данными о транскриптоме S. tuberosum. С другой стороны, только 3 % унитагов показали среднюю экспрессию выше 100 CPM, но составили 32 % от общего транскриптома. Из очень частых транскриптов только 14 % были не известны. Нужно отметить следующие транскрипты из этого исследования:

- Наиболее частый тег (StET008016) соответствует унигену TC208859, аннотированному как белок клеточной стенки – экспрессия этого гена составляла 4 % всех транскриптов в тканях листьев. Белки этого семейства выполняют функцию каркаса в качестве агглютинирующих агентов для отложения компонентов клеточной стенки (Mangeon et al., 2010). Обнаружено также их участие в защитной реакции растений против бактерий и грибов (Park et al., 2000; Fu et al., 2007).
- Через один день после инокуляции транскрипт StET009643, соответствующий гену трансальдолазы ToTAL2 (TC196885), специфически и временно повышался во время несовместимых взаимодействий. Трансальдолазы (ЕС 2.2.1.2) катализируют образование одного из предшественников шикимовой кислоты, который участвует в образовании фенилпропаноидов, алкалоидов и растительных гормонов ауксина и салициловой кислоты – важного компонента системы защиты растения
- Через три дня после инфицирования транскрипт StET010841, соответствующий гену фибриллина 8 (TC207935), был строго и специфически репрессирован. Растительные фибриллины представляют собой структурные липид-ассоциированные белки, расположенные в тилакоидных мембранах, которые, по-видимому, играют роль в реакциях биотического и абиотического стресса, роста и развития и в гормональной сигнализации.
- Снижается уровень транскриптов карбоангидразы, что может быть использовано для повышения устойчивости к P. infestans (унигены TC209461, TC218724 или TC221870 аннотированы как карбоангидразы).

В работе показано, что изменения транскрипции во время инфекции в совместимых взаимодействиях были более выраженными, чем в несовместимых. Число последовательностей-мишеней, однако, было одинаковым: 240 и 220 для несовместимых и совместимых взаимодействий соответственно. В целом транскриптомы несовместимых и совместимых взаимодействий показали больше различий, чем общности. Несовместимое взаимодействие приводит к запрограммированной гибели небольшого числа клеток в месте первичного контакта с патогеном. Транскриптом этих клеток может претерпеть значительные изменения, тогда как отобранные соседние ткани остаются относительно нетронутыми. Наблюдаемые изменения могут быть причинами или следствиями сигналов системной приобретенной резистентности.

Таким образом, через некоторое время интерес к R-генам как перспективным для селекции кандидатам существенно уменьшился, устойчивость за счет них достаточно быстро сводилась к нулю новыми расами возбудителя фитофтороза. Вместо поиска основного гена исследователи перешли к мнению, что резистентность более перспективно рассматривать как полигенный и, как следствие, количественный признак. Поэтому вектор исследований изменился на поиск новых генов-кандидатов и их картирования для эффективного сочетания аллелей повышенной резистентности в улучшенных сортах. Постулируется, что для преодоления полигенной устойчивости картофеля требуется большее число мутаций в генах авирулентности возбудителя.

При использовании SNP-маркеров были выявлены следующие гены: гены липоксигеназы (жасмонатный путь), 3-гидрокси-3-метил-глутарил-коэнзим А редуктаза (мевалонатный путь) и цитохром р450 (терпеновый биосинтез) (Mosquera et al., 2016).

С учетом предыдущих исследований (Pajerowska-Mukhtar et al., 2009; Odeny et al., 2010; Muktar et al., 2015) суммарно выявлено 10 наиболее подходящих локусов для применения в качестве диагностических маркеров в селекционных программах. Эти гены кодируют ферменты, функционирующие в жасмонатном и оксилипиновом путях (StAOS2, Plox1), в биосинтезе липидов (BCCP, биотинкарбоксильный белок-носитель) и вторичных терпеновых метаболитов (HMGCR, CYP71D11). Есть гены с неизвестной функцией (StGP28) или функцией распознавания патогенов (Rpi-vnt1) и транскрипционной регуляции (TEF1, C3HL-TF, RBP50). Их важность как генов-кандидатов для придания устойчивости в селекции важна для (1) непосредственного участия в контроле количественной устойчивости к возбудителю фитофтороза, которая не изменяется при поздней зрелости растений, (2) дальнейшей функциональной характеристики и (3) подтверждения диагностической способности в различных селекционных популяциях и средах

Изопрен синтезируется в растениях двумя путями: аце-тат/мевалонатным и дезоксиксилозафосфат/метилэритритфосфатным. Ген-участник резистентности 1-дезоксиD-ксилулозо-5-фосфат синтазы 1 (StDXS1) был обнаружен во втором пути (Henriquez et al., 2016). Его экспрессия изменяется в ответ на заражение и коррелирует с накоплением 1-дезокси-D-ксилулоза-5-фосфат синтазы – фермента, катализирующего начальную стадию 2-C-метил-Dэритрит-4-фосфатного пути (DOXP-MEP), участвующего в биосинтезе изопреноидов, необходимых для мембран хлоропластов. Изопреноиды также нужны для синтеза каротиноидов и хлорофилла, абсцизовой и гибберелиновой кислот, содержание которых, соответственно, увеличивается при увеличении экспрессии StDXS1.

Еще одна временная динамика при заражении была получена для гена StPOTHR1 с использованием трансгенных растений, где целевой ген был выключен за счет РНК интерференции (Chen et al., 2018). Белковый продукт гена StPOTHR1 локализуется на плазматической мембране клеток и существенно уменьшает степень колонизации, причем его сверхэкспрессия усиливает резистентность, экспрессироваться этот ген начинает после заражения в устойчивых сортах.

## Исследование посттрансляционных модификаций (SUMO) в процессе заражения картофеля возбудителем фитофтороза

Сумоилирование – один из типов посттрансляционной модификации белков в клетке, реализуемой за счет небольшого (~100 аминокислотных остатков) белка SUMO (small ubiquitin-related modifier), способного ковалентно присоединяться к мишени, подобно убиквитинилированию, однако не приводящему к деградации субстрата.

Инвазивные растительные патогены развили возможность модифицировать метаболизм своего хозяина, стимулируя метаболические процессы, которые способствуют росту патогена (Colignon et al., 2017). Действительно, было обнаружено, что во время процесса заражения содержание большинства известных конъюгатов SUMO S. tuberosum значительно изменяется, некоторые уменьшаются, но многие существенно увеличиваются. Выявлены белки-маркеры ответа на заражение со стороны картофеля – это патоген-связанные белки (PR1) и вышеупомянутая пластидная карбоангидраза (CA). Маркерами от P. infestans заражения были белки грибной целлюлозосинтазы (CesA3) (Grenville-Briggs et al., 2008) и гаусторий-специфический мембранный белок (PiHmp1) (Avrova et al., 2008). Синтез белка PR1 стимулируется салициловой кислотой, способность поддерживать его в высокой концентрации после заражения отличает резистентные растения (Eschen-Lippold et al., 2012). Роль CA все еще остается неясной, однако разница в уровне СА также позволяет различить устойчивые и не устойчивые к возбудителю фитофтороза сорта (Restrepo et al., 2005). Гены белковых маркеров P. infestans сначала активно экспрессировались как в устойчивых, так и в неустойчивых сортах, но при несовместимом взаимодействии их активность начинала снижаться через 24 ч после заражения. Таким образом, получены доказательства, что в восприимчивых сортах картофеля патогену удается ингибировать защитные механизмы растения и успешно инфицировать растения, в то время как в устойчивых сортах такое явление не наблюдалось.

## Заключение

В настоящее время генетических исследований защитных реакций со стороны картофеля существенно больше, чем вирулентности со стороны P. infestans, и основная их часть направлена на поиск и картирование локусов количественных признаков, отвечающих за резистентность. На заре исследований наибольший интерес вызывали R-гены, одного аллеля которых часто было достаточно для устойчивости к возбудителю фитофтороза. Таким образом, резистентность рассматривалась преимущественно как качественный признак. Позже выяснилось, что созданные сорта невыгодны с экономической точки зрения – P. infestans легко преодолевает моногенную устойчивость за счет особенностей строения своего генома. Это обстоятельство заставило пересмотреть взгляды и перейти к оценке резистентности как к признаку количественному, поэтому встал вопрос о поиске локусов количественных признаков, которые могут быть перспективны для селекции. В процессе исследования установлено, что у культурного картофеля основные локусы, дающие резистентность, часто сцеплены с негативными для продуктивности растений качествами, например с поздней зрелостью клубней. Поэтому поиск новых локусов попрежнему остается актуальной задачей.

Несмотря на большой прогресс в понимании механизмов устойчивости и способности предсказывать вспышки фитофтороза, пандемические вспышки с колоссальным уроном все еще случаются в разных странах (Fry et al., 2013; Chowdappa et al., 2015), что свидетельствует о недостаточности полученных знаний для эффективной защиты сельскохозяйственных растений и необходимости новых исследований в этом направлении.

## Conflict of interest

The authors declare no conflict of interest.
